# Unveiling the Impact of Gelation Temperature on the Rheological and Microstructural Properties of Type A Gelatin Hydrogels

**DOI:** 10.3390/polym16131842

**Published:** 2024-06-28

**Authors:** Hanaa Mehdi-Sefiani, E. Chicardi, A. Romero, Victor M. Perez-Puyana

**Affiliations:** 1 Department of Engineering and Materials Science and Transportation, University of Seville, 41092 Seville, Spain; echicardi@us.es; 2 Departamento de Ingeniería Química, Facultad de Química, Universidad de Sevilla, 41012 Sevilla, Spain; alromero@us.es

**Keywords:** gelatin-based hydrogel, gelation process, tetracycline

## Abstract

Gelatin-based hydrogels have garnered significant attention in the fields of drug delivery systems and tissue engineering owing to their biodegradability, biocompatibility, elasticity, flexibility and nontoxic nature. However, there is a lack of information regarding type-A-gelatin-based hydrogels. In this sense, the main aim of this work was the evaluation of the properties of type-A-gelatin-based hydrogel achieved using two different gelation temperatures (4 °C and 20 °C). Thus, the main novelty of this study lies in the analysis of the impact of gelation time on the rheological and microstructural properties of type-A-gelatin-based hydrogels. Moreover, the addition of a drug was also analyzed in order to evaluate the hydrogels’ behavior as a drug delivery system. For this purpose, rheological (strain, frequency sweep tests and flow curves) and microstructural (SEM) characterizations were carried out. The results demonstrated that lowering the gelation temperature improved the rheological properties of the systems, obtaining hydrogels with an elastic modulus of 20 kPa when processing at 4 °C. On the other hand, the increase in the gelation temperature improved the critical strain of the systems at low temperatures. In conclusion, this work showed the feasibility of producing hydrogels with potential application in drug delivery with different properties, varying the testing temperature and incorporating tetracycline into their formulation.

## 1. Introduction

Polymers have received meaningful attention in the vast field of biomedicine because they provide unique features of pharmacokinetics, circulation time, biocompatibility and biodegradability [1,2]. Specifically, polymeric smart materials, according to their chemical structure and composition, can react to the presence of external stimuli such as temperature, light or pH. Therefore, they can be used as materials to develop drug carrier systems, scaffolds for tissue engineering applications and sensors. Due to the variance of the temperature between the inside and outside of the human body (ranging from ambient to physiological), numerous investigations have been carried out on thermosensitive polymer hydrogels [3].

Hydrogels are three-dimensional polymeric frameworks that are crosslinked and able to absorb huge volumes of water as well as experiencing expansion and contraction [4,5]. Particularly, their porous structure and hydrophilicity render them highly appealing as biocompatible drug release systems [4,6,7,8,9]. Specifically, a drug delivery system is known as a technology, vehicle, or system that is responsible for storing and releasing an adequate dosage of the drug inside the body in a controlled and focused manner. However, apart from the properties mentioned previously, hydrogels have excellent biocompatibility and biodegradability; for this reason, hydrogels have become materials of interest for their use as drug delivery systems [10]. In fact, their three-dimensional nature and ability to retain a significant amount of water without compromising their structural integrity allow the embedding of active substances in the hydrogel network. This is achieved by mixing the substances with a monomer solution and followed by gelation. Instead of that, the active substances can also be introduced into the hydrogel network after the gelation process. This method is especially effective when those substances prevent a correct gelation process [11,12]. However, to control the loading and release rate of the substances, it is essential to maintain the porosity of the hydrogels. In this way, using hydrogels as a drug delivery vehicle offers many advantages, but they may be primarily pharmacokinetic [10]. On the other hand, hydrogels offer further engineering adaptability to improve therapeutic efficacy and maintain their structural integrity and the functionalities of embedded substances [11,12]. In fact, hydrogels can be developed in diverse physical forms such as slabs, nanoparticles, coatings, microparticles and films [10].

In general, the use of hydrogels as a drug delivery system is progressing rapidly in the direction of the development of more efficient and effective systems. Moreover, at present, the investigations about the advancement of drug delivery systems using hydrogels are focused on the reduction in systemic toxicity and the control of the release of drugs in order to release in response to a specific external stimulus such as temperature, pH, or enzymes [13]. Particularly, thermosensitive hydrogels have proven to be materials of interest in biomedical application, specifically for the development of drug carriers thanks to their excellent properties of biodegradability, low toxicity, controlled and focused drug release and high storage capacity. Specifically, thermosensitive hydrogels are interesting because their physical state changes with the temperature variation in the environment, and as a consequence, their sol–gel state also changes [14]. In fact, their swelling and gelation behaviors can be triggered by a simple temperature change [3]. Particularly, the gelation process happens due to the crosslinking of the polymer chains of the hydrogel. This crosslinking presents bonds that can be both covalent and noncovalent, such as hydrogen bonding, ionic and Van der Waals, among others. The result of this kind of crosslinking is an extensive network of interconnected polymer chains [6]. Moreover, in aquatic environments, hydrogels could be crosslinked chemically or physically through hydrophobic interactions originating from stimulation caused by the dissolution of the polymer chain in an aqueous medium. However, as the hydrogels crosslinked by chemical processes need a crosslinking agent, the crosslinked hydrogels physically present more benefits [14].

The materials that can be used for the development of hydrogels are classified into natural and synthetic categories [6]. Natural polymers present fewer toxicities when compared to synthetic ones. Additionally, natural polymers present flexibility for controlled drug release, providing benefits such as accessibility and the ability for chemical modification [1]. Hydrogels made from natural polymers can be developed using polysaccharides and proteins. The polysaccharides used can be agarose, dextran, alginate, methylcellulose, chitosan and hyaluronic acid. On the other hand, the proteins used can be fibrin and collagen [6]. Among the different natural biopolymers, collagen gains special interest due to its versatility. Thanks to the presence of this property, collagen has been used to improve biomaterials through different manufacturing processes and, therefore, give the biomaterial other final characteristics [15,16].

Collagen has shown extensive and varied versatility as a drug delivery system able to be transformed into diverse release systems after extraction from a watery solution [5]. These systems have the ability to protect the biological activity of the encapsulated molecules, resulting in optimal effects on the target cells [17].

The information about collagen has experienced exponential growth over the past few decades. As mentioned previously, collagen is a natural biopolymer whose superfamily includes 28 members that present the same structural characteristic: the presence of a triple helix that can vary from 96% (collagen I) to less than 10% (collagen XII) [18,19,20]. It is possible to obtain diverse collagen-based products with completely different structures, compositions and properties by changing the manufacturing process. As examples, we can highlight the following: soluble original collagen or undenatured original collagen (insoluble), both preserving the triple helix framework; hydrolyzed collagen (peptides/amino acids), which can be improved using various degrees of hydrolysis; and gelatin, which is denatured collagen [20].

In this study, natural polymer gelatin was chosen to develop hydrogels. Gelatins are commercially extracted from the ligaments, bones, scales, skin and tendons of bovine or porcine animals [21]. Particularly, gelatin is a natural polymer obtained through the thermal denaturation of type Ι collagen, a fibrous protein primarily found in the connective tissue of the body [18,22,23]. Its molecular weight and properties are influenced by the collagen source and the synthesis method, whether it involves enzymatic and heat denaturation or extraction under acid (type A) or alkaline (type B) conditions. The benefits of selecting gelatin as a substitute for other polymers include its intrinsic properties of being biocompatible, nontoxic, biodegradable and non-immunogenic, as well as binding moieties crucial for cell attachments [22,24]. In fact, in 2013, the use of gelatin in pharmaceutical fields increased by 21% [25]. A typical property of the gelatin solution is its ability to form hydrogels by gelation at low temperatures through cooling [23]. In particular, type A gelatin protein has been selected in this study due to its great biocompatibility and ability to form hydrogels [26].

Hydrogels based on gelatin have been considered an interesting material within the realm of tissue engineering due to their excellent properties, such as biodegradability, biocompatibility and well-accessible cell adhesion. This latter property is possible due to the ability of the gelatin to promote bioactive Arg-Gly-Asp (RGD) regions along its peptide sequence framework, mimicking the extracellular matrix and making it a highly selected biomaterial for medical use. Furthermore, gelatin-based hydrogels are desirable materials for developing drug release systems, especially for local administrations, because of their low immunogenicity and cytotoxicity levels and unique mechanical properties [27]. It is essential to highlight that the structural characteristics of the hydrogel (such as mesh size and swelling properties) and the chemical interactions between the drug molecule and the polymer matrix influence the drug release rate in hydrogels [28]. Moreover, these interactions can alter the properties of hydrogels. In fact, according to Patrick Pan, Darren Svirskis et al. [29], the interaction of the polymers with drugs can improve the bioadhesion property and enhance the local release of drugs through improved retention.

In this context, the principal objective of this research is the enhancement and characterization of type-A-gelatin-based hydrogels by studying the influence of the gelation temperature and the addition of tetracycline on their rheological and morphological properties. Furthermore, type A gelatin proteins were also characterized by means of their chemical composition and solubility.

## 2. Materials and Methods

### 2.1. Materials

Type A gelatin from porcine skin provided by Sigma Aldrich S.A. (Darmstadt, Germany) was used in this study as the primal matter to enhance the hydrogels (gel strength ca. 300). Furthermore, the solvent used was acetic acid provided by Panreac Química S.A. (Barcelona, Spain). Particularly, the acetic acid used as solvent has a concentration of 0.05 M and a pH of 3.2. Tetracycline was also used in this work and supplied by Sigma Aldrich S.A. (Darmstadt, Germany).

### 2.2. Characterization of Type A Gelatin

Type A gelatin was previously characterized by analyzing the chemical composition and its solubility as a function of pH values.

#### 2.2.1. Chemical Composition

The chemical composition of the gelatin protein was obtained following AOAC methods [30]. Briefly, protein content was assessed using a LECO CHNS-932 microanalyzer (Leco Corporation, St. Joseph, MI, USA) and by multiplying the nitrogen content by 5.95 [31]. Furthermore, lipid content was obtained using the Soxhlet method [32], and ash content was calculated by calcination at 550 °C in a muffle furnace (Hobersal HD-230, Barcelona, Spain) [33].

#### 2.2.2. Protein Solubility

Aqueous dispersions (ca. 1.00 g protein/40 mL) were prepared, and the pH of different aliquots was adjusted to alkaline pH values with 6 N NaOH and to acid pH with 2 N HCl. Samples were homogenized and subsequently centrifuged for 20 min at 10,000× *g* and 10 °C. Solubility was obtained following the Markwell method via reaction with Folin–Ciocalteu reagent and measuring the absorbance at 750 nm [33]. Solubility was expressed as a percentage (g soluble protein/100 g isolate in the sample).

#### 2.2.3. Fourier Transform Infrared Spectroscopy (FTIR)

Infrared spectroscopy was obtained with disks containing about 2 mg of sample using an FT/IR 4100 spectrophotometer (Jasco, Hachioji, Japan). Spectra were obtained for a wavenumber spectrum between 4000 cm^−1^ and 400 cm^−1^ (with an average of 200 scans and an aperture of 4 cm^−1^) and were processed with Jasco Spectra ManagerTM software (Version 2).

### 2.3. Formation of Hydrogels

The fabrication process for the type-A-gelatin-based hydrogel synthesis (3 wt.%) is detailed in Mehdi-Sefiani et al. [26]. Briefly, type A gelatin was dissolved in acetic acid (0.05 M). The solution was then centrifuged at 12,000 rpm for 7 min at 4 °C. After the centrifugation process, the tetracycline was added. Particularly, a concentration of 2.5 mg/mL of tetracycline was added to each system. Finally, the solutions were kept at 4 and 20 °C for 2 h in order to carry out the gelation process.

### 2.4. Hydrogel Characterization

To analyze the properties of gelatin-based hydrogels, a rheological and morphological characterization was carried out.

#### 2.4.1. Rheological Evaluation

In order to determine the viscoelastic characteristics of the hydrogels, an AR 2000 oscillatory rheometer (TA Instruments, New Castle, DE, USA) equipped with parallel serrated plate geometry (diameter: 40 mm) was used. Diverse studies were carried out at 5 °C (systems were named 4–5 °C and 20–5 °C for the hydrogels processed at 4 or 20 °C, respectively) and at 20 °C (systems were named 4–20 °C and 20–20 °C for the hydrogels processed at 4 or 20 °C, respectively).

- Strain sweep tests: Measurements among 0.1% and 100% strain at a consistent frequency of 1 Hz and different temperatures (5 and 20 °C) were performed to obtain the linear viscoelastic range (interval where the elastic and viscous moduli are independent of the strain applied) and the critical strain (the maximum strain supported by the sample within the linear viscoelastic range).
- Frequency sweep tests: The measurements were performed in a frequency interval between 0.02 and 20 Hz at a particular strain for each system (inside the linear viscoelastic range) and different temperatures (5 and 20 °C). In these evaluations, the elastic and viscous moduli (G′ and G″, respectively) were acquired, together with the loss tangent (tan δ = G″/G′). Furthermore, the values for G’ and tan δ at 1 Hz (G′_1_ and tan δ_1_) were selected and tabulated.
- Flow curves: These tests were performed between 0.1 and 200 s^−1^ of shear rate and at a fixed strain of 3%. These tests enable the classification of the systems as Newtonian, shear thinning or shear thickening according to the dependence of viscosity (η) on shear rate [34].

#### 2.4.2. Morphological Evaluation

The morphology of hydrogels was analyzed using a scanning electron microscope (SEM) Zeiss EVO (Jena, Germany) with a voltage of acceleration of 10 kV and a magnification of 50×. The samples were previously freeze-dried and coated with a slender layer of Au in a high-resolution sputter coater, Leica (Wetzlar, Germany).

### 2.5. Statistical Analysis

Statistical analyses were performed by means of the mean and standard deviations of the different parameters studied (*t*-tests and one-way analysis of variance, *p* < 0.05, using PASW Statistics for Windows Version18: SPSS, Chicago, IL, USA). The different measurements were performed in triplicate.

## 3. Results and Discussions

### 3.1. Characterization of Type A Gelatin

**Figure 1:**
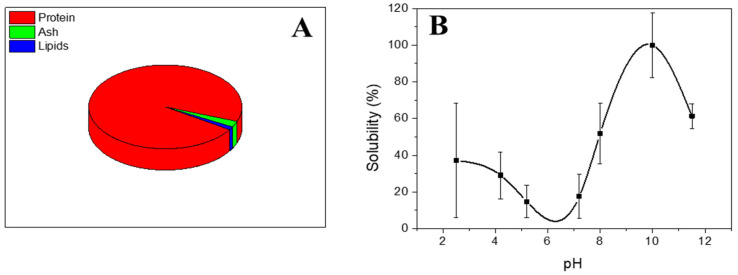
(**A**) Chemical composition and (**B**) protein solubility over the pH range studied for type A gelatin protein isolate.

The chemical composition of the type A gelatin protein is shown in Figure 1. As can be seen, gelatin protein presented a mostly protein content with a value greater than 90% by weight (specifically, it has a protein content of over 95%). Following Pearson’s classification, type A gelatin protein can be considered as a protein isolate [35]. As minor components, type A gelatin presented ash (ca. 2%) and lipids (ca. 1%). This composition is similar to the one obtained by other authors using gelatin protein as raw material [36].

Furthermore, the solubility of the protein was also studied over the pH range, and the profile obtained is also shown in Figure 1 (Figure 1B). The profile observed in the solubility curve shows a sinusoidal profile with initial values around 40% for acidic pHs around 2–4. A decrease in solubility then occurs in the pH range around pH 6 and pH 7.5–8. This decrease in solubility coincides with the point of minimum solubility of the type A gelatin protein. In other words, type A gelatin protein presents its isoelectric point in the pH range between 6.5 and 8. Next, an increase in the solubility of the protein is observed, obtaining values greater than 60% at pH 8 and even reaching 100% at basic pH such as pH 10. Finally, there is a slight decrease in solubility (standing at around 70% for pH 11). Comparing the solubility profile to previous profiles obtained in recent works, it can be confirmed that a similar isoelectric point was obtained to the one obtained by other authors for gelatin protein [37,38].

**Figure 2:**
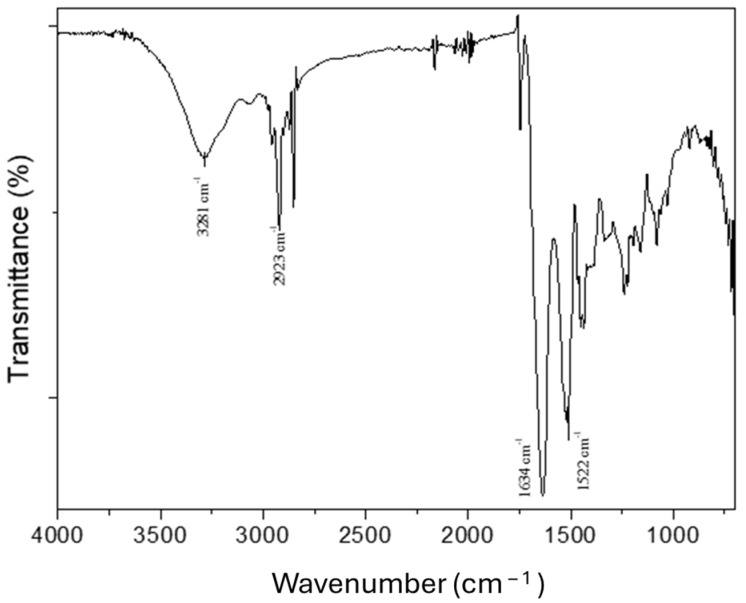
Fourier Transform Infrared Spectroscopy (FTIR) of the type A gelatin protein isolate.

Apart from the chemical composition and solubility of the type A gelatin protein, its chemical interactions have also been studied using Fourier Transform Infrared Spectroscopy (FTIR), and the results are shown in Figure 2. Particularly, the peaks of the FTIR informed us about the stretching and bending of the bounds. In the frequency range between 3300 and 3200 cm^−1^, a signal called Amide A is produced because of the stretching of the NH bound. On the other hand, in a frequency range between 2940 and 2850 cm^−1^, the presence of another signal called Amide B is indicated, which is associated with the symmetrical stretching of CH_2_. Furthermore, the stretching of C=O, which produces the absorption of the Amide I band, occurs in the range 1700–1600 cm^−1^. Finally, if the N-H bending occurs in the range between 1550 and 1520 cm^−1^, this indicates the presence of the Amide II signal; however, if the N-H bending occurs in the range 1245–1235 cm^−1^, it indicates the presence of the Amide III signal [39].

### 3.2. Characterization of Gelatin-Based Hydrogels

As known, in this study, hydrogels were prepared using two different temperatures (4 °C and 20 °C). At this point, it is important to highlight that temperature influences the speed at which water molecules move unidirectionally through the nanopores of the hydrogel, which could consequently affect the physically crosslinked three-dimensional network. Thus, the crosslinked structures of hydrogels could have some peculiarities that are strongly associated with the preparation temperature. Most likely, these peculiarities could be related to the size of the crosslinking points as well as the density of the crosslinking for the entire hydrogel network [40].

#### 3.2.1. Effect of the Gelation Temperature

Firstly, strain sweep tests were carried out (Figure S1), and the resulting critical strain values are reported in Table 1. At this point, it is important to highlight two characteristics: on the one hand, the crosslinking density and, on the other hand, the presence of the microcrystalline domains in hydrogels, which are composed of densely folded chains and act as inherent phases with high energy. Moreover, these domains restrain the degrees of freedom of movement of the well-aligned chains. The presence of these domains is important because the water weakens polymer–polymer interactions [41].

Attending to the results shown in Table 1, more deformable systems are obtained by increasing the gelation temperature. This is because as previously mentioned, the temperature alters the speed at which water molecules flow unidirectionally through the nanopores, giving rise to mobility between protein chains [40]. Thus, according to Feng-Ya Jing et al. [40], in such a state, segments of chains between crosslinking points experience a micro-Brownian movement whose intensity increases with increasing temperature, achieving higher flexibility. On the other hand, Feng-Ya Jing et al. [40] reported that at a higher temperature of treatment, the presence of microcrystalline domains decreases, which also provides a higher flexibility to the system.

**Figure 3:**
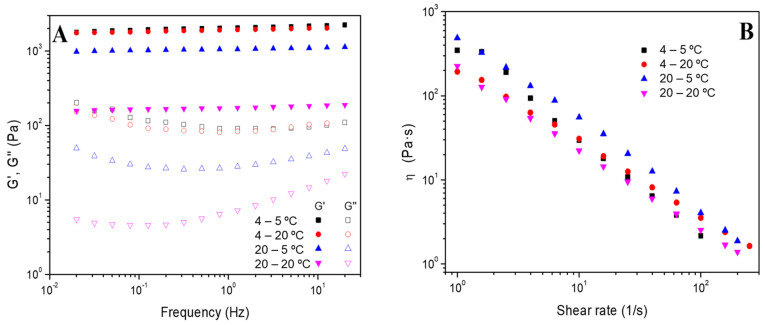
(**A**) Frequency sweep tests and (**B**) flow curves of the gelatin-based hydrogels processed at 4 °C and 20 °C and measured at 5 °C and 20 °C (hydrogels named 4–5 °C, 4–20 °C, 20–5 °C and 20–20 °C, respectively).

The frequency sweep tests of the different systems processed at 4 °C (hydrogels 4–5 °C and 4–20 °C) and 20 °C (hydrogels 20–5 °C and 20–20 °C) were also performed, and Figure 3A shows the results obtained. The gelation process does not affect the stability of the gelatin-based hydrogels since a similar constant profile is obtained. Nevertheless, a slight decrease in G’ values was obtained by increasing the gelation temperature (from 4 to 20 °C). Therefore, a lower gelation temperature raised the solid nature of the hydrogels. However, the effect of the measuring temperature depends on the processing temperature of the hydrogels.

It can be highlighted that the values of the hydrogels labeled as 4–5 °C and 4–20 °C are quite similar, while the hydrogels labeled as 20–5 °C and 20–20 °C are not. These results could be attributed to the fact that the hydrogels prepared at 4 °C are more stable than those whose gelation temperature was 20 °C. Thus, despite changing the processed temperature at which the rheology study was carried out, its elastic and viscous moduli were not altered.

Furthermore, flow curves were also obtained to study the systems’ viscosity and their progress with the shear rate. The results shown in Figure 3B revealed that the hydrogels presented a shear-thinning behavior since viscosity decreases as the shear rate increases. In this sense, the structure of all hydrogels is very sensitive to shear without significant differences in their behavior.

**Figure 4:**
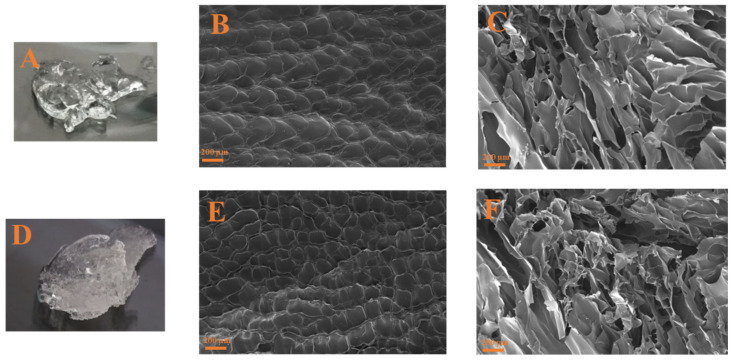
Superficial and cross-sectional SEM images of the gelatin-based hydrogels processed without tetracycline ((**B**) and (**C**), respectively) and with tetracycline ((**E**) and (**F**), respectively). Macroscopic images of the hydrogels without (**A**) and with (**D**) tetracycline are also included.

In addition to the mechanical characteristics, SEM imaging of the hydrogels was obtained. Hydrogels processed without tetracycline presented a similar microstructure as the one shown in Figure 4 (Figure 4B,C). The surface (Figure 4B) shows a homogeneous and uniform distribution, similar to the one obtained with tetracycline (Figure 4E), whereas the cross-sectional image (Figure 4C) exhibited a typical laminar structure, characteristic of gelatin-based hydrogels [42,43]. Moreover, it can be observed that hydrogels with tetracycline (Figure 4F) had pore sizes lower than hydrogels without tetracycline (Figure 4C). According to the results obtained for critical strain and the microstructural characterization, it could be considered that the hydrogels are more stable when their pores are smaller and more compact.

#### 3.2.2. Effect of the Addition of Tetracycline

**Figure 5:**
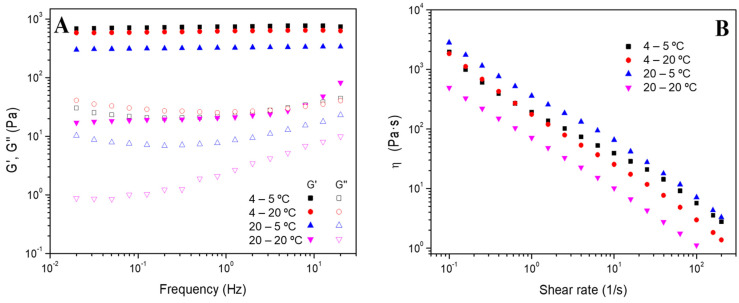
(**A**) Frequency sweep tests and (**B**) flow curves of the gelatin-based hydrogels processed at 4 °C and 20 °C (hydrogels named 4–5 °C, 4–20 °C, 20–5 °C and 20–20 °C) with tetracycline.

Apart from studying the influence of the gelation temperature, the possible modifications suffered by the hydrogels with the addition of a drug to be potentially released were also analyzed. Thus, tetracycline was added to the initial formulation, and the mechanical and morphological properties were evaluated. In this sense, Table 1 shows how, as a general trend, the addition of tetracycline increased the critical strain of the hydrogels. This effect indicated that when temperature remains constant, the presence of tetracycline incremented the molecular chains per unit volume and generated a higher stable network of links for water entry and impairment of the hydrogel [44]. Moreover, if we focused on the numerical value, it can be noted that the hydrogels fabricated at 4 °C showed a greater increase in critical strain. According to Meng Ru et al. [44], this phenomenon can be attributed to a more concentrated packing mode caused by the increase in the triple helix ratio and the chain aggregation center. Furthermore, the frequency sweep tests (Figure 5A) showed that G’ values are mostly higher than G” values, which indicates that the elastic behavior predominates. However, it can be noted that the temperature of the test still alters the properties of the hydrogel. Comparing the systems with and without tetracycline (Table 2), the addition of tetracycline significantly decreased the rheological properties of both hydrogels produced at either 4 or 20 °C, although the addition of tetracycline does not significantly influence the evolution or the values of viscosity of the systems.

On the other hand, the addition of tetracycline induced slight modifications in the microstructure of the hydrogels, as shown by the cross-sectional images of the hydrogels without and with tetracycline in the formulation (Figure 4C and Figure 4F, respectively). Nevertheless, the typical laminar structure of gelatin-based hydrogels is maintained independently of the addition of tetracycline (Figure 4B,D).

## 4. Conclusions

Hydrogels were successfully developed at two different gelation temperatures, 4 and 20 °C. Firstly, the characterization of the raw material, type A gelatin, revealed the presence of 95% protein and an isoelectric point in the range of pH 6.5–8.

Regarding the hydrogel characterization, the results of rheological measurements showed that the critical strain of the hydrogels increased with the presence of tetracycline and decreased with a lower gelation temperature. On the other hand, the measuring temperature had a greater effect on the G’ of hydrogels with a higher gelation temperature due to possible mobility between the protein chains. Moreover, the addition of tetracycline significantly decreased the rheological properties of both hydrogels produced at either 4 or 20 °C, making the system less solid. However, the addition of tetracycline did not influence either the evolution or the values of viscosity of the systems.

Finally, the microstructural characterization of the selected systems showed that the hydrogel with and without tetracycline presented a porous structure. Specifically, the hydrogel without tetracycline had higher pore-size values, indicating a slightly greater character.

For future research, gelatin obtained from biowastes could be used as raw material for the development of different hydrogels in order to analyze their properties for being used as drug delivery carriers of different active pharmaceutical ingredients. Moreover, it is also possible to study the crosslinking of gelatin with different agents as well as the use of hybrid hydrogels.

## Figures and Tables

**Table 1:** Critical strain (%) at different temperatures (5 °C and 20 °C) for the hydrogels according to the gelation temperature (4 °C and 20 °C) and the presence of tetracycline. Different letters were incorporated as exponents to indicate meaningful distinctions between the values.

SYSTEMS	Critical Strain (%)	
	−	+ Tetracycline
Hydrogel 4–5 °C	32.4 ± 10.3 ^a^	102.7 ± 0.2 ^d^
Hydrogel 4–20 °C	14.8 ± 0.3 ^b^	99.9 ± 0.1 ^e^
Hydrogel 20–5 °C	104.4 ± 0.5 ^c^	103.7 ± 0.7 ^cf^
Hydrogel 20–20 °C	9.2 ± 6.7 ^b^	13.0 ± 3.0 ^b^

**Table 2:** G′ and tan δ at 1 Hz (named G′_1_ and tan δ_1_) obtained at different temperatures for the hydrogels according to the gelation temperature (hydrogels named 4–5 °C, 4–20 °C, 20–5 °C and 20–20 °C) and the presence of tetracycline (+tetracycline). Different letters are incorporated as exponents to indicate meaningful distinctions between the values per parameter.

Systems	G′_1_ (Pa)		tan δ_1_ (−)	
	−	+Tetracycline	−	+Tetracycline
Hydrogel 4–5 °C	1983 ± 91 ^a^	692 ± 42 ^d^	0.04 ± 0.01 ^A^	0.03 ± 0.01 ^A^
Hydrogel 4–20 °C	1928 ± 86 ^a^	518 ± 95 ^c^	0.03 ± 0.01 ^A^	0.09 ± 0.01 ^B^
Hydrogel 20–5 °C	1044 ± 1 ^b^	371 ± 52 ^c^	0.03 ± 0.01 ^A^	0.02 ± 0.01 ^A^
Hydrogel 20–20 °C	300 ± 101 ^c^	20 ± 5 ^e^	0.03 ± 0.01 ^A^	0.04 ± 0.01 ^A^

## Data Availability

The data presented in this study are available on request from the corresponding author. The data are not publicly available due to the data also form part of an ongoing study.

## Supplementary Materials

The following supporting information can be downloaded at: https://www.mdpi.com/article/10.3390/polym16131842/s1, Figure S1. Strain sweep tests of of the gelatin-based hydrogels processed at 4 °C and 20 °C and measured at 5 °C and 20 °C (hydrogels named as 4–5 °C, 4–20 °C, 20–5 °C and 20–20 °C, respectively) (A) without and (B) with tetracycline.
