# Supplementary material for: Unveiling the Impact of Gelation Temperature on the Rheological and Microstructural Properties of Type A Gelatin Hydrogels
Source: Polymers (Basel). 2024 Jun 28;16(13):1842. doi: 10.3390/polym16131842 (PMC11243819; doi:10.3390/polym16131842)
Supplement: Supplementary file 1 [file polymers-16-01842-s001.zip › polymers-3053747-supplementary.pdf]

Unveiling the Impact of Gelation Temperature on the Rheological and Microstructural Properties of Type A Gelatin Hydrogels

Hanaa Mehdi-Sefiani [1,2,*], E. Chicardi [1], A. Romero [2] and Victor M. Perez-Puyana [2,*]

[1] Department of Engineering and Materials Science and Transportation, University of Seville, 41092 Seville, Spain
[2] Departamento de Ingeniería Química, Facultad de Química, Universidad de Sevilla, 41012 Sevilla, Spain
* Correspondence: hmehdi@us.es (H.M.-S.); vperez11@us.es (V.M.P.-P.)

SUPPLEMENTARY MATERIAL

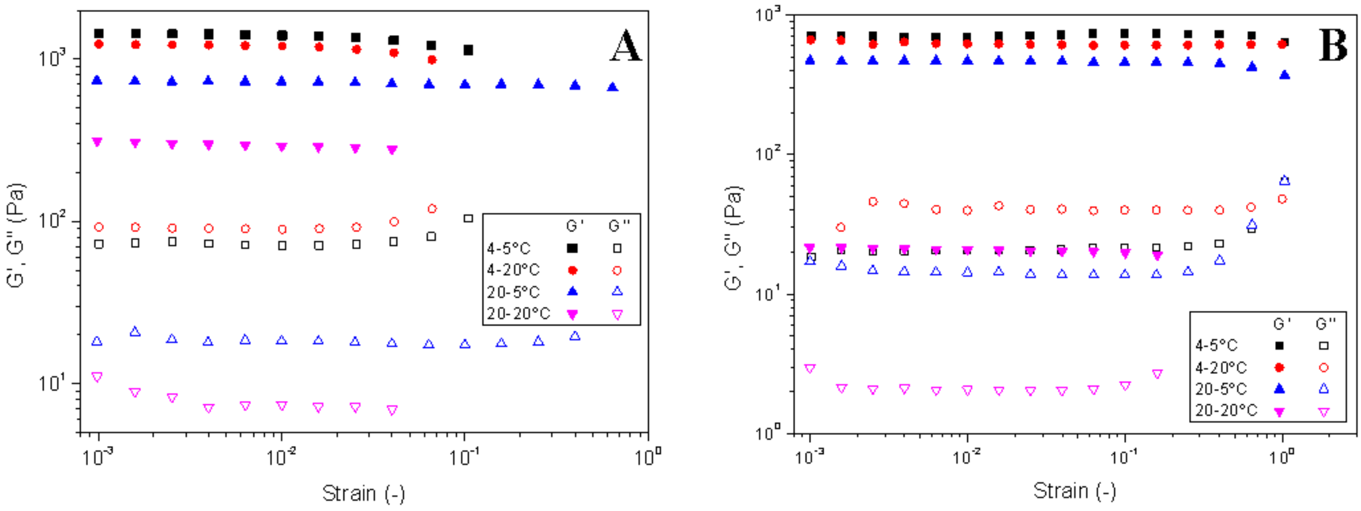

Figure S1. Strain sweep tests of of the gelatin-based hydrogels processed at 4 ºC and 20 ºC and measured at 5 ºC and 20 ºC (hydrogels named as 4-5ºC, 4-20ºC, 20-5ºC and 20-20ºC, respectively) (A) without and (B) with tetracycline.
